# Biochemical and structural characterization of a novel halotolerant cellulase from soil metagenome

**DOI:** 10.1038/srep39634

**Published:** 2016-12-23

**Authors:** Roma Garg, Ritika Srivastava, Vijaya Brahma, Lata Verma, Subramanian Karthikeyan, Girish Sahni

**Affiliations:** 1 CSIR-Institute Of Microbial Technology, Council Of Scientific and Industrial Research (CSIR), Sector 39 A, Chandigarh 160036, India

## Abstract

Cellulase catalyzes the hydrolysis of β-1,4-linkages of cellulose to produce industrially relevant monomeric subunits. Cellulases find their applications in pulp and paper, laundry, food and feed, textile, brewing industry and in biofuel production. These industries always have great demand for cellulases that can work efficiently even in harsh conditions such as high salt, heat, and acidic environments. While, cellulases with high thermal and acidic stability are already in use, existence of a high halotolerant cellulase is still elusive. Here, we report a novel cellulase Cel5R, obtained from soil metagenome that shows high halotolerance and thermal stability. The biochemical and functional characterization of Cel5R revealed its endoglucanase activity and high halostability. In addition, the crystal structure of Cel5R determined at 2.2 Å resolution reveals a large number of acidic residues on the surface of the protein that contribute to the halophilic nature of this enzyme. Moreover, we demonstrate that the four free and non-conserved cysteine residues (C65, C90, C231 and C273) contributes to the thermal stability of Cel5R by alanine scanning experiments. Thus, the newly identified endoglucanase Cel5R is a promising candidate for various industrial applications.

Cellulase is extensively used in various bio-ventures such as pulp and paper, textile, laundry, food and feed, brewing and agricultural industries^1^,^2^. Moreover, increasing societal demand on rapidly depleting fossil fuels as principal energy source and its consequent environmental effects has necessitated the development of alternative energy sources. The production of renewable bio-fuels using naturally abundant lignocellulosic biomass such as agricultural and forestry wastes will alleviate the dependence on fossil fuels^3^. A major component of these biomass wastes is cellulose and hence the obvious choice as a promising and efficient source of biofuel^4^,^5^,^6^. The biochemical conversion of lignocellulose to ethanol involves three steps: first, pretreatment of biomass to remove lignin and hemicellulose, second, enzymatic hydrolysis of the cellulose and third, fermentation of glucose to produce ethanol^7^. The pretreatment of biomass usually occurs at high temperature in the presence of acids or bases; the neutralization of these acids and bases results in the formation of salts^8^. These salts need to be removed, which consume tons of water and energy, for further downstream processes. Therefore, enzymes that are stable in the presence of salts or tolerant to them are in great demand during downstream processes. Thus, for reasons of stability and catalytic activity, vigorous search is on to identify novel and highly efficient cellulases that are suitable for industrial production and consumer affordability^1^,^6^. Cellulases belong to glycosyl hydrolase family of enzymes (including endo-, exo-glucanase and β-glucosidase) which catalyze the degradation of cellulose into glucose monomer units (cellulolysis) in a concerted manner. Endoglucanase (EC 3.2.1.4) randomly cleaves internal β-1,4-glucan linkages, producing free ends. Exoglucanase (EC 3.2.1.91 and 3.2.1.176) progressively acts on reducing and non-reducing ends to release the cellobiose moieties. The di-saccharide thus produced is further digested by β-glucosidases (EC 3.2.1.21) to release free glucose in a catalytic manner^9^. These enzymes work synergistically to bring about efficient cellulose hydrolysis^10^,^11^. Endoglucanases are major enzyme groups that initiate the hydrolysis of internal linkages. According to CAZy (Carbohydrate-Active enZYmes) database classification, endoglucanases are very diverse and are part of 14 glycosyl hydrolase (GH) families^12^. Among the known strategies^13^,^14^,^15^, metagenomics (culture independent approach) is a unique way to access the hidden information in unexplored microbial lineages and discover novel genes, metabolic pathways, and industrially important products^16^,^17^ as only 0.1–1% of the microbes are culturable under laboratory conditions.

In this study, we report a novel endoglucanase, Cel5R, that belong to GH5 family, identified by soil metagenomic approach, which is tolerant to high salt conditions with moderate tolerance to temperature and pH. In addition, we describe the sequence analysis, cloning, soluble expression, purification, biochemical and structural characterization of Cel5R. The Cel5R shows thermostability up to 58 °C and pH stability from 5–9. Surprisingly, the Cel5R shows halotolerance and extreme halostability in 4 M NaCl, 3 M LiCl and 2 M KCl which is higher than other known halostable cellulases^18^,^19^. Thus, the combination of extreme halostability with moderate thermal and pH stability makes Cel5R a potential candidate for industrial applications.

## Results

### Identification of a novel cellulase from soil plasmid library

A metagenomic library was constructed in pEZSeq vector using the DNA directly isolated from the soil. The library had the average insert size of 2–5 kb. Functional screening of the library on LB plates containing 0.5% CMC (Carboxymethyl cellulose) revealed a positive clone with an insert size of ~5 kb that showed a clear zone of hydrolysis on CMC plate. The plasmid DNA was isolated from the positive colony and subsequently sequenced by primer walking. The sequence analysis showed the presence of a gene cluster of 5553 bases consisting of several open reading frames (ORFs) (Supplementary Fig. S1). The different ORF’s along with the most probable hits and their accession numbers are shown in Supplementary Table S1. The ORF showing putative cellulase (c*el5Rα*) of size 1014 bp encodes for a 338 amino acid residue protein belonging to the glycosyl hydrolase family 5, subfamily 2 (GH5_2) according to CAZy (Carbohydrate Active enZYmes) database classification^20^. The BLAST search using the nucleotide sequence of *cel5Rα* revealed about 65-70% identity (up to 30% query coverage) with other known cellulases. However, the closest endoglucanase from *Paludibacter propionicigenes* which showed 68% identity with c*el5Rα* has not been characterized yet. Similarly, the BLAST search using the deduced amino acid sequence of c*el5Rα* revealed about 40–70% identity (up to 90% query coverage) with that of known cellulases and closest being endoglucanase from *Paludibacter jiangxiensis*, (69% identity; 81% similarity) which is also not yet been characterized. Pfam database predicted a conserved domain in Cel5Rα belonging to GH5 family of cellulase. Phylogenetic tree analysis also indicated that the *cel5Rα* ORF belongs to GH5 family of cellulase and clustered with the three main anaerobic cellulolytic organisms *Paludibacter, Prevotella buccae* and *Bacteroides* sps. (Supplementary Fig. S2). Multiple sequence alignment by ClustalW^21^ revealed that the active site residues of GH5 endoglucanases were all conserved in Cel5Rα (Supplementary Fig. S3). The molecular mass and isoelectric point (pI) of full length polypeptide sequence was estimated to be 38662.5 Daltons and 4.86, respectively.

### Expression and purification of the recombinant endoglucanase

**Figure 1:**
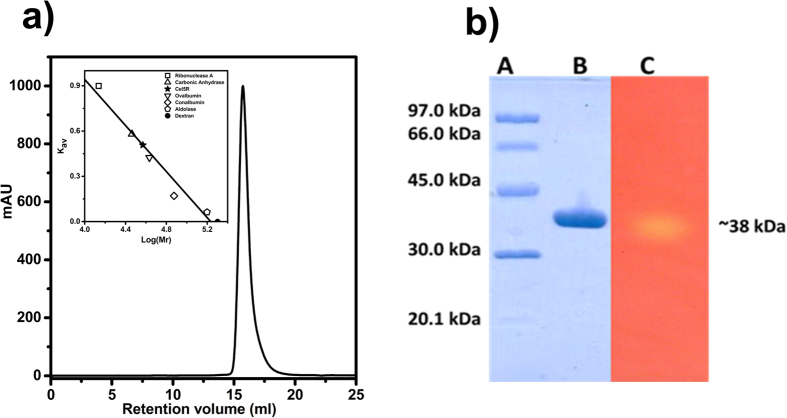
Purification of endoglucanase Cel5R. (**a**) Gel filtration profile of Cel5R confirms the monomeric (38 kDa) nature of the protein. The inset shows calibration plot of standards: ribonuclease (13.7 kDa), carbonic anhydrase (29 kDa), ovalbumin (43 kDa), conalbumin (73 kDa), aldolase (158 kDa) and dextran (2000 kDa), (GE) run on the same column. (**b**) SDS-PAGE profile and Zymography of endoglucanase Cel5R, Lane A, Molecular marker, Lane B, GFC purified Cel5R, Lane C, Zymogram analysis to detect the activity based identity of Cel5R.

Initially, the *cel5Rα* was cloned in pET15b vector with N-terminal His-tag and the protein expression was checked in *E. coli* Rosetta (DE3) cells. The Cel5Rα protein was found to be expressed in insoluble fraction (data not shown) which was confirmed by SDS-PAGE analysis. However, when the crude cell lysate from *E. coli* Rosetta (DE3) cells harboring *pET15b-cel5Rα* was incubated onto the well bored in LA-CMC plate along with the empty vector cell lysate (negative control), a clear zone of hydrolysis was visible around the well after staining with congo red. This result indicated that Cel5Rα encodes for cellulase with CMCase (Carboxymethyl cellulase) activity (data not shown), although, its expression in *E. coli* cells was found in inclusion bodies. It is known in literature that the removal of the hydrophobic signal peptide can increase the expression and solubility of the recombinant protein without altering the biochemical and functional properties^22^. The sequence analysis of Cel5Rα by SignalP 4.1 revealed the presence of N-terminal signal peptide with the cleavage site between the Thr-27 and Glu-28 residues. Accordingly, the initial N-terminal 27 amino residues were removed from Cel5Rα to create Cel5R. The expression of Cel5R in *E. coli* resulted in higher levels of protein in soluble form. The size of the protein was confirmed on 10% SDS-PAGE which showed an over-expressed protein band close to 38 kDa. Two step purification using Ni-NTA affinity chromatography followed by gel permeation chromatography of over-expressed protein resulted in pure monomeric population of Cel5R (Fig. 1a). Zymography also clearly exhibited a single band of activity against the expected size of 38 kDa (Fig. 1b) confirming the correct size and active form of Cel5R.

### Biochemical characterization of Cel5R

**Figure 2:**
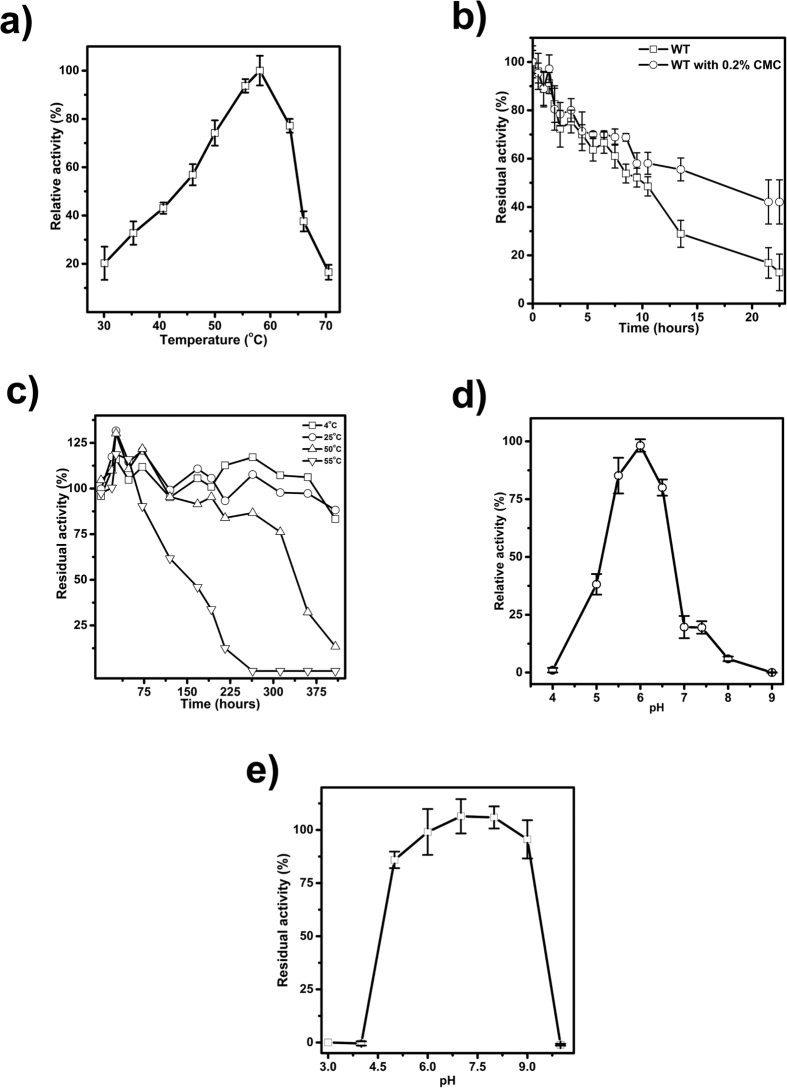
Stability characterization of metagenome derived novel endoglucanase Cel5R. (**a**) The temperature optima profile for endoglucanase showed that Cel5R exhibited maximum activity at 58 °C. (**b**) Effect of substrate on the thermostability was established by incubating enzyme at 58 °C in the presence and absence of 0.2% substrate (CMC). (**c**) Residual activity of Cel5R monitored at optimum pH at various temperatures and at different intervals of time representing the thermal stability (**d**) pH profile of Cel5R showing maximum activity at pH 6.0. (**e**) Residual activity of Cel5R after incubating Cel5R at different pH for seven days.

The enzyme activity of Cel5R and other biochemical and kinetic parameters were determined by DNS (3,5-Dinitrosalicylic acid) method using CMC as substrate. The optimal temperature for the enzyme activity was found to be 58 °C (Fig. 2a) with half-life period of about 10 hours. However, at optimum temperature, the half-life of Cel5R was enhanced to 16 hours in the presence of 0.2% CMC (Fig. 2b). Moreover, the Cel5R kept at 4 °C and 25 °C was stable for several days without much loss in activity while at 50 °C and 55 °C its half-life was found to be 340 hours and 150 hours respectively, which revealed its thermostable behavior (Fig. 2c).

While, Cel5R showed catalytic activity in the pH range of 5.0-6.5, the highest catalytic activity was observed at pH 6.0 in 100 mM sodium-citrate buffer (Fig. 2d). In addition, Cel5R was stable over a relatively broad pH range i.e. between pH 5–9, as it retained 80–100% activity after 7 days of incubation at room temperature (Fig. 2e). At pH 4, the enzyme retained 50% hydrolytic activity after 24 hours of incubation at room temperature (data not shown). Differential effect of sodium-acetate and sodium-citrate buffer of same pH value and strength on activity (two fold higher activity in citrate buffer) was also observed (data not shown) which may be due to the difference in adsorption of anions on the Cel5R molecule leading to aggregation or unfolding^23^. Moreover, the kinetic studies with different concentration of substrate (CMC) revealed a typical Michaelis-Menten behavior of Cel5R with K_m_ and V_max_ value of 5 mg/ml and 312 U/mg respectively.

The activity profiling of Cel5R on various substrates suggested that it was very specific to β-1,4-glucosidic linkages of CMC (220 ± 9 U/mg) and mixed β-1,4 and β-1,3-glucosidic linkages of barley-β-glucan (435 ± 10 U/mg). It also cleaved the agluconic β-D-cellobioside linkage in *p*NPC (1.85 U/mg) but was not active on *p*NPG indicating its endo-mode of action. Also Cel5R could not hydrolyze laminarin which has β-1,3 linkages and displayed no activity on xylan, starch and locust bean gum (Table 1). Insoluble crystalline substrates like avicel and filter paper were also resistant to Cel5R activity but phosphoric acid swollen cellulose (PASC) which was swollen amorphous form of avicel provided the sites for Cel5R hydrolysis. PASC is generally composed of cellulose II form which is accepted as the model for naturally occurring amorphous cellulose^24^,^25^. Cel5R displayed 1.5 ± 0.5 U/mg on PASC which may vary from different lots of substrate due to its heterogeneity. Thus, Cel5R is a novel endoglucanase with a high specific activity on soluble substrates as well as insoluble amorphous PASC.

**Figure 3:**
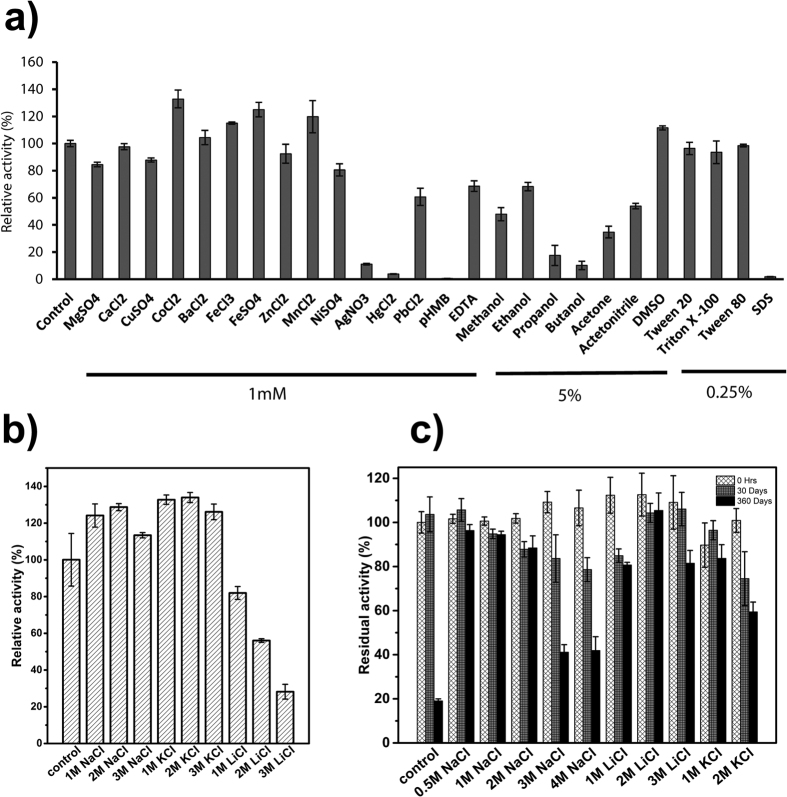
Enzymatic activity in presence of various solutes. (**a**) Effect of various metal salts (1 mM), organic acids (5%) and detergents (0.25%) on Cel5R activity. (**b**) Relative activity of Cel5R in the presence of different concentrations of salts (NaCl, LiCl and KCl). (**c**) Residual activity of Cel5R at zero time point, after incubation for one month and one year in different concentrations of salts showing its halostable nature. The activity of Cel5R in the absence of any solute was taken as 100%.

The activity of Cel5R was tested in the presence of various metal ion salts. Most of them had no effect on activity while 1 mM of CoCl_2_, FeSO_4,_ MnCl_2_ and FeCl_3_ enhanced the activity slightly. Cel5R is not a metallo-enzyme as EDTA did not inhibit its activity completely. Cel5R had significant activity in the presence of methanol and ethanol at 5% concentration while propanol and butanol had diminishing effect. The DMSO enhanced the Cel5R enzymatic activity by about 10%. Detergents (Tween 20, Tween 80, Triton X-100) tested at 0.25% concentration had little effect while SDS completely abolished the activity of Cel5R (Fig. 3a). Also, Cel5R was completely inhibited by 1 mM AgNO_3_, HgCl_2_, and *p*-(Hydroxymercuri)benzoic acid (pHMB) indicating that thiols might play role in catalysis^26^ (Fig. 3a).

### Cel5R shows high halotolerant and halostability

Interestingly, we observed that the hydrolytic activity of Cel5R was enhanced on increasing the salt concentration in the assay. There was about 24%, 28% and 13% enhancement in the catalytic activity of Cel5R in the presence of 1 M, 2 M and 3 M NaCl respectively (Fig. 3b). Likewise, 1 M, 2 M and 3 M concentrations of KCl also had almost 30% enhancing effect (Fig. 3b). On the other hand lithium ion (Li^+^) had diminishing effect on activity probably due to its high hydration energy which may lead to distortion of water structure around the macromolecule^27^. Moreover, Cel5R showed a remarkable stability in the presence of 4 M NaCl, 3 M LiCl and 2 M KCl on prolonged incubation for 30 days at room temperature (Fig. 3c). The Cel5R retained 100% activity in the presence of 3 M LiCl, 75–80% activity in 4 M NaCl, and 70-80% activity in 2 M KCl which categorized it as an extreme halostable cellulase^28^,^29^. Surprisingly, Cel5R retained 70–100% activity when incubated for one year in the presence of salts 2 M NaCl, 3 M LiCl and 1 M KCl (Fig. 3c).

### Role of cysteines in Cel5R stability

**Figure 4:**
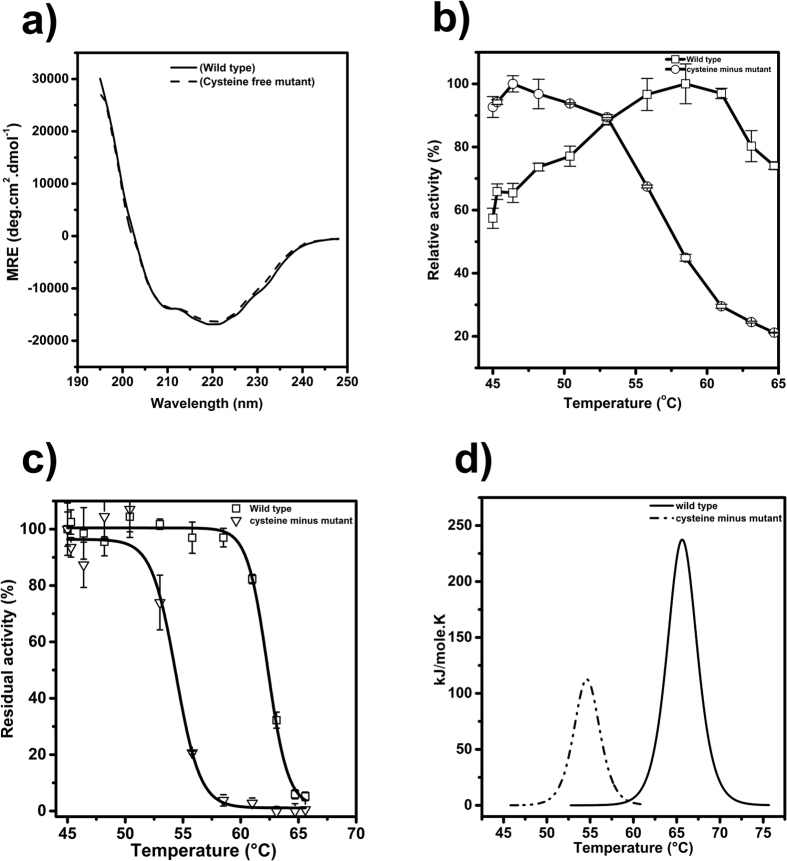
Role of cysteine residues in Cel5R thermostability. (**a**) Circular Dichroism of the wild type and cysteine free mutant of Cel5R showed similar secondary structure content of both proteins. (**b**) Replacing all cysteines with alanine shifted the temperature optima from 58 °C to 46 °C, establishing the role of cysteines in maintaining thermostability of Cel5R. (**c**) Thermal inactivation curve was obtained by measuring residual enzyme activity of Cel5R and its cysteine free mutant after incubating them at different temperatures for 10 minutes. (**d**) Thermal unfolding curves of wild type and cysteine free mutant by DSC showed their melting temperatures at 65 °C and 55 °C respectively.

The DTNB (5,5′-dithiobis-[2-nitrobenzoic acid]) assay under denaturing conditions confirmed the presence of four free cysteine residues which are not conserved as seen by multiple sequence alignment (Supplementary Fig. S3). The observed near-complete inhibition of endoglucanase activity by thiol inhibitors led us to investigate the role of free cysteines in the activity of Cel5R. Moreover, it has been shown that the substitution of free thiols with other amino acid residues increased^30^ the thermal stability of the protein in some cases, while in others it is decreased^31^. Thus, to understand the role of cysteines in Cel5R, different constructs with single, double and quadruple mutations (cysteine to alanine) were made and their activities were checked by DNS (3,5-Dinitrosalicylic acid) assay. Though most of the single (C65A, C90A, C231A, C273A) and double mutants (C65A/C90A, C65A/C231A, C65A/C273A C90A/C231A, C90A/C273A, C231A/C273A) had lesser or comparable activity to wild type Cel5R, the cysteine free mutant retained only 20% activity (Table 2). However, the major elements of secondary structure contents remained same in wild type and cysteine free mutant as confirmed by CD experiments (Fig. 4a). As previously discussed, the wild type Cel5R was inactivated by Hg^2+^, Ag^+^ and pHMB. Similar to wild-type, the Hg^2+^ also inhibited the activity of cysteine free mutant of Cel5R. This may be due to binding of Hg^2+^ with other residue such as tryptophan which is shown to be essential for substrate binding in GH5 family^32^. However, unlike the wild-type, the pHMB did not affect the activity of cysteine-free mutant of Cel5R indicating pHMB may bind to thiols as the inhibition of Cel5R was reversed in the presence of DTT (data not shown).

In addition, the temperature optima of cysteine-free mutant (46 °C) shifted towards lower temperature compared to wild type (58 °C) (Fig. 4b). The cysteine-free mutant was also thermally less stable as shown by thermal inactivation curve (Fig. 4c). The thermal unfolding experiment study by DSC (Differential Scanning Calorimetry) showed that the melting temperature was shifted by 10 °C between wild type (Tm- 65 °C) and cysteine-free mutant (Tm- 55 °C) (Fig. 4d). Notably, the melting temperature of single and double mutants was lower than the wild type Cel5R except C273A which had Tm slightly higher than wild type (Table 2). Thus, mutating all cysteines to alanine drastically reduced the thermo-stability of Cel5R, indicating the role of cysteines in maintaining the native-like structure and stability.

### Overall structure and active site of Cel5R

**Figure 5:**
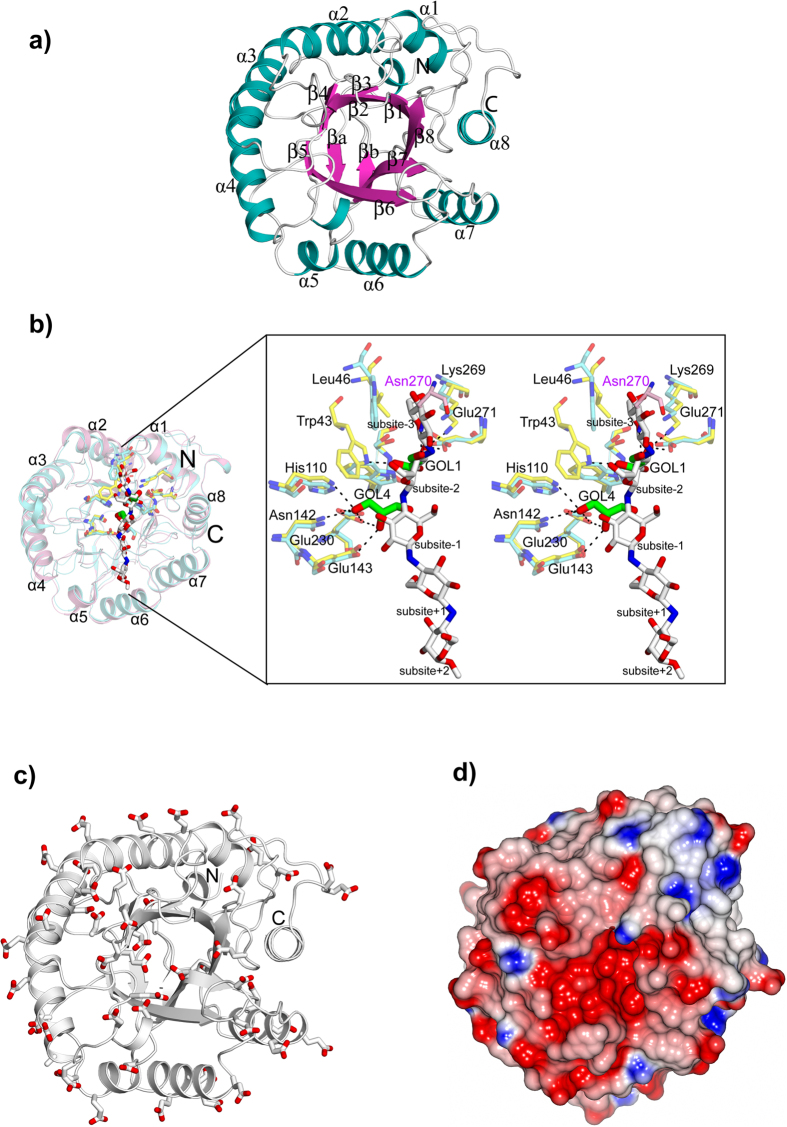
Crystal structure of endoglucanase Cel5R. (**a**) Cartoon diagram showing the C^α^ trace of Cel5R. Cel5R showed the presence of TIM barrel fold common to GH5 cellulases. The 8 parallel β-strands forming a β-barrel structure is shown in magenta. The 8 α-helices surrounding the β-barrel is shown in cyan. (**b**) The superposition of Cel5R (pink) with Cel5A endoglucanase (cyan) of *B. agaradhaerens* bound with thiopentasaccharide (white). The inset shows the stereo diagram of proposed active site residues of Cel5R (shown in sticks) superposed on to the catalytic site of Cel5A endoglucanase along with thiopentasaccharide ligand. The hydrogen bond interactions are shown in dotted lines. The residue Asn270 from other Cel5R monomer is shown in magenta. (**c**) The location of the Asp and Glu residues in Cel5R are shown in sticks. The figures were generated through PyMOL^80^. (**d**) Electrostatic surface representation of atoms of Cel5R. The negative charges are shown in red, positive charges are shown in blue and neutral charges are shown in white. This figure was generated using CCP4MG^81^.

To understand the molecular mechanism of Cel5R, its crystal structure was determined by molecular replacement method at 2.2 Å resolution. The crystal belonged to P2_1_2_1_2_1_ space group, and consists of two Cel5R molecules in an asymmetric unit. The results of PISA^33^ server did not indicate any stable interaction at the protein-protein interface, thus eliminating the possible existence of Cel5R as dimer in solution. This result was also consistent with analytical size-exclusion chromatography studies where Cel5R eluted as a monomer. The PDBeFOLD^34^ server predicted Cel5A from *Baciilus agaradhaerens* (PDB id: 1QI2) to be the closest structural homolog with root mean square deviation (rmsd) of 0.86 Å over 293 C^α^ atoms. The overall structure of Cel5R was similar to other members of the GH5 family, and consists of (β/α)_8_ – barrel fold, commonly known as the TIM barrel (Fig. 5a). Along with canonical TIM barrel fold, the structure had two extra β-strands running antiparallel to each other at the N-terminus. The two antiparallel β-strands are labelled as βa and βb in Fig. 5a and the secondary structure elements are arranged in the order (βa-βb-β1-α1-β2-α2-β3-α3-β4-α4-β5-α5-β6-α6-β7-α7-β8-α8).

### Catalytic site of Cel5R

To identify the catalytic site of Cel5R, we superimposed its structure with other known GH5 family cellulase structures such as *B. agaradhaerens* (PDB ID: 1QI2, rmsd 0.9 Å for 293 C^α^ atoms; PDB ID: 1H5V, rmsd 1.0 Å for 293 C^α^ atoms) and *Bacillus sp*. (PDB ID: 1G0C, rmsd 1.4 Å for 291 C^α^ atoms) that were bound with the substrates. The superposition of these structures revealed that the residues forming the catalytic site were well conserved in Cel5R suggesting it may display a similar catalytic mechanism^35^. It is known that the hydrolysis of glycosidic bond is carried out by general acid catalysis which requires two vital residues that act as a proton donor and nucleophile/base^36^. Moreover, depending on the distance between the two vital residues, the hydrolysis of glycosidic bond may procced with a mechanism of either overall retention or an inversion of anomeric configuration^36^. The enzyme with retaining mechanism shows an average distance of 5.5 Å between the two catalytic residues while it is about 10 Å for inverting enzyme^36^. The superposition of Cel5R with other cellulases indicated that the residues Glu143 and Glu230 were likely to be two critical residues, where Glu143 acts as a proton donor while Glu230 acts as a nucleophile. In Cel5R the distance between Glu143 and Glu230 was found to be 6.2 Å suggesting it may follow retaining mechanism. In addition, to identify the residues involved in substrate binding we superimposed the *B. agaradhaerens* GH5 (1H5V) structure complexed with glucose units on to the Cel5R crystal structure^37^ (Fig. 5b). In 1H5V, the active site was bound with five glucose units and located at −3, −2, −1, +1 and +2 subsite positions respectively. The superimposition revealed that, in Cel5R, the subsite −3 was occupied by Asn270 from the other monomer of Cel5R. In addition, the stacking interaction provided by the Trp39 residue with the glucose molecule at −3 subsite was missing in Cel5R as the corresponding residue was replaced by Leu46. The substitution of Trp to Leu at the catalytic site had been shown to play a role in substrate binding^38^,^39^. In Cel5R, the subsites −2 and −1 were occupied by glycerol molecules (Fig. 5b). The cis peptide bond formed between Trp264-Ser265 in Cel5R (Trp262–Ser263 in case of 1H5V) was conserved and this Trp residue provided the hydrogen bond interaction at subsite −2. The glycerol molecule positioned at −2 subsite in Cel5R, interacted with Trp43 through its O1 while its O2 interacted with Lys 269 and Glu 271. Similarly, the glycerol molecule in Cel5R close to −1 subsite interacted with His110, Asn 142, Glu 230 and Glu 143. The residues which were expected to form interactions at +1 and +2 subsites were also conserved in Cel5R.

Despite such a striking similarity with other non-halophilic GH5 structures, the high halotolerance and halostability showed by Cel5R was surprising. Although, literature survey indicated that the structural determinants for the halotolerance of the enzyme is still elusive, a consensus based on the analysis of different halotolerant/halophilic proteins suggested that they tend to possess more acidic residues on the surface of the protein than their non-halophilic homologs^40^. Specifically, both Asp and Glu residues on the surface have been shown to contribute significantly towards their halotolerance^40^. Interestingly, the analysis of amino acid sequence of the Cel5R and its structure revealed that there were 52 acidic residues (Asp + Glu; 16.7%) present on its surface which were relatively higher than other halophilic cellulases reported till now.

## Discussion

Metagenomics has become an important tool to explore the science behind ‘unculturables’. Cellulose degradation, an important step in several industries, is carried out by series of enzymes acting synergistically to bring the complete hydrolysis of cellulose. The functional screening of a soil metagenomic library led to the identification of a gene (*cel5Rα*) with endoglucanase activity. The heterologous bacterial expression of full-length Cel5Rα resulted in inclusion bodies formation while the removal of N-terminal hydrophobic signal peptide increased the expression and solubility of the recombinant protein (Cel5R) without altering its properties.

The Cel5R has high optimal working temperature of 58 °C and is also very stable at this temperature with half-life period of about 10 hours, which classifies Cel5R as a thermostable enzyme^41^. This optimum temperature is comparable to BsCel5A cellulase that was isolated from *Bacillus subtilis* 168 (T_opt_-60 °C) and CelI15 from *Bacillus subtilis* I15 (T_opt_-60 °C) which are close structural homologue of Cel5R^42^,^43^. However, the optimum temperature of Cel5R is much higher than other reported thermostable cellulases isolated from *Bacillus* sp. KSM-S237 (T_opt_-45 °C) and *Bacillus* strain C1 (T_opt_-50 °C)^44^,^45^. Notably, the thermostability is enhanced when Cel5R is incubated in the presence 0.2% CMC at 58 °C which could be due to stabilization provided by the hydrolyzed products to the active site^46^.

Cel5R shows catalytic activity in the pH range of 5.0–6.5. This is similar to the already reported BsCel5A from *Bacillus subtilis* 168, a structural homologue of Cel5R^42^. On the other hand, Cel5A from *Baciilus agaradhaerens*, another structural homologue of Cel5R, is an alkaliphilic cellulase and becomes inactive at low pH^47^. Cel5R is also stable over a relatively broad pH range i.e. pH 5–9 for seven days at room temperature. Thus Cel5R can tolerate both acidic as well as basic pH range. A recently published report on the acid stable endoxyloglucanse showed pH stability in the range of 3.5–7 for only 24 hours^48^. In other report, acid-stable cellulase derived from a metagenome retained about 80% of maximum activity from pH 5 to 9 for only 16 hours^49^.

We have observed that the catalytic activity of Cel5R was inhibited by thiol reagents such as pHMB and Hg^2+^ suggesting that cysteines might play a role in catalysis. However, biochemical and structural characterization have revealed that all the cysteine residues in Cel5R exist in reduced form and they are not part of the catalytic site. Therefore, to understand the contribution of cysteine residues in the catalysis of Cel5R, they were substituted to alanine, which is the least destabilizing substitution for cysteine^50^. Interestingly, while the single and double mutants of Cel5R have lesser or comparable catalytic activity to that of wild type, the cysteine free mutant retained only 20% catalytic activity to that of wild type (Table 2) indicating that the free cysteines might play a role in catalysis. In addition, a large body of published reports shows that free cysteine residues have stabilizing effect and renders thermostability to the protein^51^. However, in some cases the free cysteines are reactive and unstable and their replacement with other amino acid resulted in increased thermostability of the protein^52^. Thus, to understand the contribution of free cysteines in the thermostability, we measured the melting temperature of wild type and cysteine mutants of Cel5R. The wild type, single and double mutants of Cel5R show comparable thermostability while it is decreased significantly for the mutant devoid of all cysteines (Table 2). This is in contrast to the previous report where the removal of free cysteines improved the thermotolerance of Cel6A^53^. The crystal structure analysis indicates that the free cysteines in Cel5R are involved in hydrogen bond interaction with the neighboring residues that are participating in catalysis (interactions between Cys65 with Leu59, Cys90 with Phe86, Cys231 with Val262 and Csy273 with Gly238, Glu271). The mutation of free cysteine residues may perturb these hydrogen bonds and possibly the van der Waals interactions, causing reduced catalytic activity and thermostability of Cel5R. A similar reduction in catalysis and thermostability is also observed in family 11 xylanase^54^. Taken together we show that the free cysteines in Cel5R play a role both in catalytic activity and thermostability although they are not part of the active site.

In addition to thermostability and pH stability, halotolerance and extreme halostability shown by Cel5R suggested that the gene might belong to a halophilic organism, but it is not possible to determine the organism to which it belonged. When the activity was checked after one year of prolonged incubation with high salt conditions, it was observed that the presence of salts (2 M NaCl, LiCl and KCl) conferred stability to Cel5R compared to control reaction where no salt was present. Moreover, Cel5R shows activity in the presence of high salt concentration. Recently, it has been reported that the halophilic cellulase isolated from Icelandic hot spring showed decreased activity in the presence of increasing concentration of NaCl compared to control reaction with no salt^19^. On the other hand, the thermophilic GH5 endoglucanase isolated from *Thermoanaerobacter tengcongensis* MB4 retains less than 15% of its activity after 12-hours of pre-incubation in 4 M NaCl^55^. The enzyme isolated by Voget *et al*. retained 86% activity after incubation with 3 M NaCl, 3 M RbCl or 4 M KCl for 20 h^56^. However, Cel5R shows extreme halostability for a longer duration as compared to the previous published reports. The halotolerance arises due to the presence of acidic residues (Asp and Glu) on the surface of protein and halophilic proteins have large number of charged surface residues than their non-halophilic counterparts^40^. In fact, the mutation of surface residues in malate dehydrogenase from *H. marismortui*^57^ and glucose dehydrogenase from *H. mediterranei*^58^ affected only the halophilic properties of mutant without affecting the kinetic parameters and enzymatic activity of the protein. The crystal structure analysis also reveals that the halophilic nature shown by Cel5R may be due to acidic residues (16.7% with 52 residues) present on the surface of the protein (Fig. 5c and d). In contrast, the endoglucanase from *Bacillus subtilis 168* (PDBID: 3PZT) has only 38 (11.6%) acidic residues^42^ and the recently discovered GH5 cellulase from *Thermoanaerobacterium* which is also shown to be halostable cellulase has only 43 (11.3%) acidic residues present in it^19^. Thus, based on these observations, we speculate that the halotolerant ability shown by the Cel5R is due to large number of acidic residues (Asp + Glu) present on the surface of the protein. This property makes Cel5R, ideal, to be used in various industrial processes where concentrated salt solutions formed after pretreatment and neutralization of biomass would otherwise inhibit enzymatic conversions^59^. Thus, Cel5R is an example of extreme halotolerant cellulase despite being the fact that it is isolated from moderate environment.

## Materials and Methods

### Materials

Soil DNA isolation kits UltraClean and PowerMax were procured from Mo Bio Laboratories Inc., Carlsbad, CA, USA. The vector pEZSeq for library construction was purchased from Lucigen Corporation, Middleton, USA. The T7 promoter-based expression vector, pET15b and *E. coli* strains (XL1B and BL21) were procured from Novagen Inc. (Madison, Wisconsin). DNA amplification and modifying enzymes such as *Pfu* DNA Polymerase, Restriction Endonucleases, T4 DNA ligase, *Dpn*I were obtained from New England Biolabs (NEB, USA). Phusion polymerase was procured from Thermo Fisher Scientific, USA. Oligonucleotides were synthesized from Integrated DNA Technologies (IDT, USA). PCR/plasmid purification kits and Ni-NTA resin were procured from Qiagen (Germany). HiLoad 16/60 Superdex 75 and Superdex 200 10/300 GL column used for gel filtration chromatography was purchased from GE-Amersham Biosciences. The substrates carboxymethyl cellulose (CMC), *para*-nitro phenyl cellobioside (*p*NPC), barley-β-glucan, avicel, laminarin etc. were procured from Sigma-Aldrich (USA). All reagents used in the experiments were of high quality grade available.

## Methods

### Sample collection, construction and screening of metagenome libraries

The soil sample was collected at the depth of 5 cm from the outer region of Institute of Microbial Technology, Chandigarh (30.7478°N, 76.7337°E). The metagenomic DNA was isolated directly from the soil using commercially available UltraClean and PowerMax kits. The isolated DNA was partially digested with Sau3AI followed by ligation in blunt end cloning vector pEZSeq and transformed in XL1B cells. The clones obtained were screened on Luria-Bertani agar plates containing 0.5% CMC as substrate. After overnight incubation, the plates were stained with 0.2% congo red for 15 minutes and then destained with 1 M NaCl followed by visualization of yellow zone of hydrolysis around the colony^60^. The plasmid was extracted from the clone that was showing CMCase activity.

### Sequence analysis

The plasmid from the positive clones was sequenced by primer walking approach. ORFs were predicted using ORF finder (http://www.ncbi.nlm.nih.gov/projects/gorf/) and annotated based on the conserved domains present in them^61^. The protein sequence for the ORF encoding cellulase was derived using ExPASy translate tool and other parameters like the molecular mass and *pI* of the encoded protein were estimated using ExPASy protparam tool^62^. Sequence similarity was assessed by NCBI BLAST program^63^. Signal peptide sequence was predicted by using Signal P 4.1 server^64^. The active site residues and conserved domain were predicted by Pfam database^65^. To find out the conserved regions and residues, the deduced amino acid sequence of cellulase encoding ORF was subjected to NCBI BLASTP search against PDB (Protein Data Bank) database^61^ and non-redundant top hits were aligned using ClustalW^66^ module of BioEdit software^67^.

Phylogenetic tree was constructed by Neighbor-Joining method^68^. The non-redundant protein sequences obtained by NCBI BLASTX^61^ analysis were aligned using the ClustalW^66^. The resulted aligned sequences were used in MEGA 6.06^69^ for the construction of unrooted phylogenetic tree by Neighbor-joining method. One thousand bootstrap replications and Poisson corrections were carried out for assuring statistical confidence.

### Construction of recombinant plasmid

The ORF encoding endoglucanase gene, named as c*el5Rα*, was PCR amplified using primers cel5R_F with *Nde*I site and cel5R_R with *Bam*HI site (Supplementary Table S2) and pEZSeq-Cel as template. The amplified product was digested and cloned in *Nde*I and *Bam*HI sites of pET15b vector with N-terminal 6X His-tag sequence (*pET15b-cel5Rα*). In the same way, *pET15b-cel5R* (the N-terminal truncated version of cel5Rα) was also constructed by PCR amplification using cel5RΔ27_F (Supplementary Table S2) primer with *Nde*I site and cel5R_R with *Bam*HI site using *pET15b-cel5Rα* as template. The amplified PCR product was cloned in similarly digested pET15b vector with N-terminal 6X-His tag. The sequence and in-frame integrity of the clones were confirmed by automated DNA sequencing on Applied Biosystems 3130xl Genetic Analyzer 16 capillary DNA Sequencer.

### Protein expression and purification

The plasmid *pET15b-cel5R* was transformed in *E. coli* Rosetta (DE3) cells. A single colony carrying the plasmid construct was grown in Luria-Broth media containing 100 μg/ml of ampicillin at 37 °C with shaking at 200 rpm. The overnight grown culture was inoculated in fresh LB media (supplemented with 100 μg/ml ampicillin) and the expression was induced with 0.5 mM IPTG (Isopropyl β-D-thiogalactopyranoside) after OD_600_ reached to 0.6 AU. After 5 hours of post-induction incubation, cells were harvested, resuspended in lysis buffer (20 mM phosphate buffer, pH 7.4, 300 mM NaCl, 1 mM phenylmethylsulfonyl fluoride (PMSF) and then sonicated with 30 seconds on and off pulse for half an hour (Sonics, Vibracell, USA). The lysate was centrifuged for 15,000 *g* for 20 minutes and the protein expression profile of induced versus uninduced culture was checked on 10% SDS-PAGE. The cellulase activity of Cel5Rα was confirmed on LA-CMC (0.5% CMC) plate.

For Cel5R purification, the *E. coli* Rosetta (DE3) cells harboring pET15b-cel5R were grown as described above. The harvested cells were resuspended in equilibration buffer (20 mM potassium-phosphate buffer pH 7.4, 300 mM NaCl, 1 mM PMSF, 10 mM imidazole) and sonicated with 30 sec on and off cycle for 30 minutes (Sonics, Vibracell). The crude lysate was pelleted down by centrifugation at 15,000 *g* for 30 minutes and the supernatant was loaded onto a pre-equilibrated Ni-NTA affinity column (GE Healthcare). Column washing was done using the buffer containing 20 mM phosphate buffer pH 7.4, 300 mM NaCl and 30 mM imidazole, subsequently protein was eluted by increasing imidazole concentration to 300 mM in the buffer. The eluted protein was subjected to overnight dialysis against 20 mM phosphate buffer pH 7.4, 10% glycerol, 300 mM NaCl. Dialyzed protein was concentrated using Amicon ultra centrifugal filters (Merck, Darmstadt, Germany) and subjected to gel filtration chromatography on HiLoad16/60 Superdex75 column (GE Healthcare), pre equilibrated with 20 mM phosphate buffer pH 7.4 and 300 mM NaCl. The purity and integrity of the protein was estimated by SDS-PAGE analysis. Zymography was performed according to the protocol described by Choi^70^.

The oligomeric nature of the protein was estimated using Superdex 200 10/300 GL column which was calibrated with low molecular weight calibration standards (GE Healthcare). The molecular weight of Cel5R was determined using the calibration curve (plot of log M_r_ versus K_av_) of the standards. K_av_ and M_r_ denote the gel phase distribution coefficient and molecular weight respectively.

### Enzyme characterization and cellulase activity

The hydrolytic activity of the enzyme was checked using DNS assay^71^ which measures the reducing sugar units released by hydrolysis of polysaccharide. One unit (U) is defined as the quantity of enzyme required to release reducing sugar at micromoles (μmoles) per minute rate. The reaction mixture contained 1% (w/v) CMC and 30–40 ng of purified Cel5R in 100 mM buffer in a total volume of 60 μl. Reactions were incubated in Eppendorf Master cycler for 15 minutes and stopped using 60 μl of DNS reagent. It was further incubated at 95 °C for 5 minutes for color development and absorbance at 540 nm was measured. Optimal pH and temperature conditions were determined in 100 mM of different buffers from pH range of 4–9 and temperature ranging from 30–70 °C respectively. The buffers used were sodium-citrate (pH 4–6), Tris-Cl (pH 7–8) and Glycine/NaOH (pH 9). Thermal stability was determined by incubating Cel5R in 100 mM of citrate buffer, pH 6 at various temperatures (4 °C, 25 °C, 50 °C, 55 °C, 58 °C) and checking the residual activity at various times under standard reaction conditions. Thermal stability in the presence of substrate (0.2% w/v CMC) was checked by incubating enzyme at 58 °C and measuring the residual activity under standard reaction conditions. pH stability was checked by incubating enzyme in 100 mM buffers with different pH at 25 °C and then checking the residual activity under optimal conditions after regular time intervals. For thermal inactivation, enzyme was incubated at various temperatures (45–65 °C) for 10 minutes and the residual activities were checked by performing activity assay at the optimal condition. Kinetic parameters (K_*m*_, V_*max*_) were calculated under optimal conditions with 40 ng enzyme and substrate concentrations ranging from 1.6 mg/ml to 18.33 mg/ml of low viscosity Na-CMC.

The substrate specificity was checked by using 1% (w/v) of different substrates (Avicel, filter paper, barley-β-glucan, locust bean gum, laminarin, xylan and Na-CMC) in assays performed under standard reaction conditions within dynamic range of activity. Phosphoric acid swollen cellulose (PASC) was prepared as described^25^ and its concentration was determined to be 7 mg/ml. Activity on PASC was determined in the reaction containing 60 μl of enzyme with 60 μl of PASC (7 mg/ml) in 100 mM Na-Citrate buffer (pH 6) and incubation at 58 °C for 1 hour. The reaction was stopped by addition of DNS as described earlier. The hydrolyzed products released can be quantitatively estimated by FACE (Fluorescence-assisted carbohydrate electrophoresis)^72^. The activity on *para*-nitrophenyl-β-D-cellobioside and *para*-nitrophenyl-β-D-glucopyranoside was checked by incubating 50 μl of 10 mM substrate with 50 μl (0.2 μg) of diluted enzyme for 15 minutes at 58 °C. The reaction was terminated with 100 μl of 1 M Na_2_CO_3_ and OD at 405 nm was recorded (One unit is defined as the quantity of enzyme required to release 1 μmole of *para*-nitrophenol per minute). The effect of various metal ions (Mg^2+^, Ca^2+^, Cu^2+^, Co^2+^, Ba^2+^, Fe^2+^, Zn^2+^, Mn^2+^, Ni^2+^, Ag^2+^, Hg^2+^, Pb^2+^) and chelating agent EDTA was probed using 1 mM concentration of each in the reaction mixture. The effect of detergents (Tween-20, Triton X-100, Tween 80, sodium dodecyl sulphate) and organic solvents (methanol, ethanol, propanol, butanol, acetone, acetonitrile, dimethyl sulphoxide (DMSO)) were tested at 0.25% and 5% (v/v) concentration respectively. Halotolerance of Cel5R was determined by measuring the activity in the presence of 1–3 M sodium chloride (NaCl), Lithium chloride (LiCl) and potassium chloride (KCl). Halostability was checked by incubating the enzyme in presence of different concentrations of salts for various intervals of time and then measuring the residual activity under standard conditions.

### Circular Dichroism analysis of the protein

Far-UV circular dichroism (CD) spectra of protein at 5 μM (10 mM phosphate buffer, pH 7.4 at 25 °C) was collected using Jasco J-810 spectro polarimeter (Jasco International Co., Japan) in the range of 195–250 nm using 1 mm quartz cuvette. Results have been expressed as mean residual ellipticity (deg.cm^2^.dmol^−1^). A total of 3 spectra were collected which were averaged and corrected by subtraction of the blank.

### DTNB assay to measure the free thiols

DTNB or Ellman’s reagent, measures free thiols present in protein^73^. The amount of free thiols was calculated using the molar extinction coefficient of 2-nitro-5-thiobenzoic acid dianion (TNB^−2^) as 13600 M^−1^cm^−1^ and measuring absorbance of protein sample at 412 nm against the known concentration of protein. Sulphydryl group was quantitated using β-mercapto-ethanol (single thiol) as standard. The Ellman’s reagent (1 mM) was allowed to react with protein/standard in TE buffer (100 mM Tris-Cl (pH 8), 1 mM EDTA) containing 2% SDS at room temperature for 15 minutes, and then absorbance at 412 nm was recorded.

### Construction of cysteine mutants and their activity

Single site cysteine to alanine mutations were performed using High fidelity Phusion polymerase kit (Thermo Fisher scientific, US). Complementary primers with the desired mutations (Supplementary Table S2) were designed and extended by Phusion polymerase in the temperature cycler. The PCR products were digested with DpnI enzyme and transformed in *E. Coli* XL1 Blue cells. After sequence confirmation, the cloned plasmids were transformed in expression host *E. Coli* Rosetta (DE3) cells. The mutant proteins were purified following the same protocol mentioned above and the activity was checked by DNSA method.

### Differential Scanning Calorimetry (DSC)

The melting temperature (Tm) of the proteins was determined on Nano-DSC (TA Instruments-Waters LLC, New Castle, DE). Cel5R and its mutants were dialysed in 20 mM phosphate buffer (pH 7.4) and used at 1 mg/ml for calorimetry experiment. The samples were scanned at 1 °C/minute between temperatures 25–80 °C and data was analysed using NanoAnalyse software.

### Crystallization of endoglucanase Cel5R

Crystallization was carried out using the concentrated protein of Cel5R (40 mg/ml in 20 mM Tris pH 7.5, 100 mM NaCl and 20% glycerol). The initial crystallization screens were set in 96 well plate (Molecular Dimensions Ltd, UK) by mixing 1 μl of protein and 1 μl of precipitant solution and incubated at 20 °C. Cel5R crystals appeared next day in several conditions of Index screen (Hampton Research, USA). Further optimization of these conditions were performed using sitting drop method with 2 μl of protein and 2 μl of precipitant equilibrated against 200 μl reservoir solution in a 48 well plate. After optimization, 0.2 M Magnesium chloride hexahydrate, 0.1 M Tris pH 8.5, 25% PEG 3350 was found to be suitable for obtaining diffraction quality Cel5R crystals.

### Data collection and processing of Cel5R

The X-ray intensity data for Cel5R crystal was collected using an in-house MAR345dtb image plate detector mounted on a Rigaku Micromax-007 HF rotating anode X-ray generator that was operated at 40 KV and 30 mA. The crystal was briefly soaked in reservoir solution containing 20% glycerol as cryoprotectant prior to data collection. A total of 167 images were collected at the wavelength of 1.542 Å. Each image was exposed for 5 minutes with 1° oscillation. The X-ray intensity data were collected up to 2.2 Å and the data set was indexed, integrated, scaled using XDS suite^74^ of programs and merged using AIMLESS^75^ as implemented in CCP4^76^. The Cel5R crystal was crystallized in orthorhombic space group P2_1_2_1_2_1_ with unit cell parameters a = 45.77, b = 88.13, c = 146.47 Å.

### Structure determination and refinement

The structure of Cel5R was solved by molecular replacement method using PHASER^77^ as implemented in CCP4. The endo-1,4-beta-glucanase of *Bacillus subtilis* 168 (PDB ID: 3PZT, 64% sequence similarity) was used as a search model. The PHASER with default parameters gave a single solution with two molecules of Cel5R in the asymmetric unit. The initial model was refined with rigid body refinement using REFMAC5^78^ and iterative rounds of model building and restrained refinement were carried using COOT^79^ and REFMAC5 respectively until model was built completely. The data collection and refinement statistics are shown in Table 3.

### Nucleotide sequence accession number

Nucleotide sequence encoding the endoglucanase was deposited at GenBank database under the accession number AND74761.

### PDB ID

The atomic coordinates and structure factors for Cel5R have been deposited in protein data bank (PDB) (http://wwpdb.org/) with PDB ID 5I2U.

## Additional Information

**How to cite this article**: Garg, R. *et al*. Biochemical and structural characterization of a novel halotolerant cellulase from soil metagenome. *Sci. Rep.* **6**, 39634; doi: 10.1038/srep39634 (2016).

**Publisher's note:** Springer Nature remains neutral with regard to jurisdictional claims in published maps and institutional affiliations.

## Supplementary Material

Supplementary Information

## Figures and Tables

**Table 1:** Substrate specificity of Cel5R.

Substrate	Linkage type	U/mg
Na-CMC	β-1,4-glucan	220 ± 9
Locust bean gum	α-1,6/, β -1,4-galatomannan	UD*
Oat spelt xylan	β-1,4-xyloglucan	UD*
Laminarin	β-1,3/1,6-glucan	UD*
Barley beta glucan	β-1,3/1,4-glucan	435 ± 10
Avicel/ filter paper	β-1,4-glucan	UD*
PASC	β-1,4-glucan	1.5 ± 0.2

^*^UD- undefined.

**Table 2:** Relative activity of various cysteine to alanine mutants and their melting temperatures.

Mutant	Relative activity (%)	Melting temperature (°C)
WT	100 ± 1.836	65.99
C65A	87.85 ± 1.17	65.1
C90A	78.96 ± 5.322	63.5
C231A	74.46 ± 3.02	64.5
C273A	106.91 ± 3.4	67.1
C65A C90A	73.63 ± 1.73	60.7
C65A C231A	72.94 ± 1.86	63.8
C65A C273A	109.71 ± 0.53	65.4
C90A C231A	61.46 ± 3.87	61.4
C90A C273A	62.93 ± 2.62	63.5
C231A C273A	70.92 ± 1.04	64.4
C65A C90A C231A C273A	19.24 ± 1.78	55

**Table 3:** Data collection and refinement statistics.

Particulars	Endoglucanase Cel5R
**Data collection details**	
Wavelength (Å)	1.5418
Resolution range (Å)	45.77-2.20 (2.27–2.20)^a^
Space group	P2_1_2_1_2_1_
Unit cell parameters (Å)	a = 45.77, b = 88.13, c = 146.47
Total number of reflections	180959
Unique reflections	30246
Average mosaicity (°)	0.64
Redundancy	6.0 (5.1)
Mean I/σ (I)	15.9 (2.7)
Completeness (%)	98.3 (88.0)
R_merge_ (%)^b^	7.8 (45.7)
**Refinement details**	
Resolution range (Å)	45.77 - 2.20
R_cryst_ (%)^c^	20.9
R_free_ (%)^d^	25.7
**RMS deviations**	
Bond length (Å)	0.009
Bond angle (°)	1.30
No. of residues in Chain A/B	296/297 (out of 312)
No. of solvent molecules	134
No. of magnesium/phosphate ions	2/2
No. of glycerol molecules	9
**Ramachandran plot, residues in**	
Most favoured region (%)	94.53
Additionally allowed region (%)	4.62
Outliers (%)	0.85
**Average B-factor (Å**^**2**^)	
From Wilson Plot	36.0
For chain A/B	39.3/37.6
For solvent molecules	36.3
For magnesium/phosphate ions	38.3/73.0
For glycerol molecules	46.1
**PDBID**	**5I2U**

^a^Values for the last resolution shell are in parentheses.

^b^R_merge_ = ∑_*hkl*_∑_*i*_│*I*_*i*_(*hkl*) − *I(hkl*)│/∑_*hkl*_∑_*i*_*I*_*i*_(*hkl*) where *I(hkl*) is the intensity of reflection *hkl*.

^c^R_cryst_ = ∑_*hkl*_∥*F*_*obs*_│ − │*F*_*calc*_∥/∑│*F*_*obs*_│.

^d^R_free_ is the cross-validated R_cryst_ factor computed for the test set of 5% of unique reflections.
