# Supplementary material for: Biochemical and structural characterization of a novel halotolerant cellulase from soil metagenome
Source: Sci Rep. 2016 Dec 23;6:39634. doi: 10.1038/srep39634 (PMC5180356; doi:10.1038/srep39634)
Supplement: Supplementary Information [file srep39634-s1.doc]

**Supplementary data:**

**BIOCHEMICAL AND STRUCTURAL CHARACTERIZATION OF A NOVEL HALOTOLERANT CELLULASE FROM SOIL METAGENOME**

ROMA GARG, RITIKA SRIVASTAVA, VIJAYA BRAHMA, LATA VERMA, SUBRAMANIAN KARTHIKEYAN AND GIRISH SAHNI*


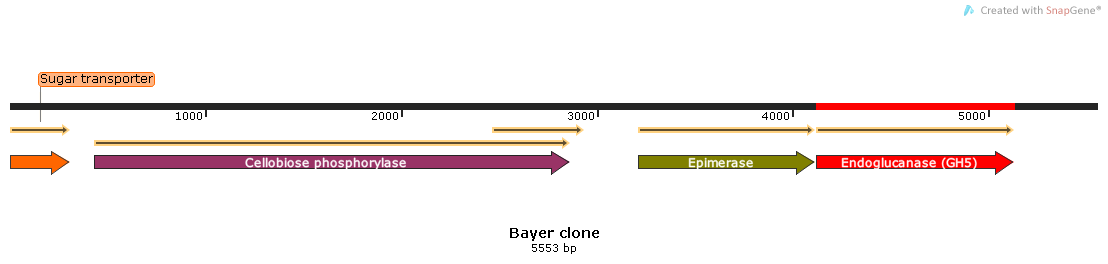


**Fig S1**. **Schematic diagram showing sequence analysis of positive plasmid clone**. The presence of different ORFs in the stretch of 5553 bp DNA fragment along with the most probable hits obtained by BLAST. The ORF belonging to cellulase is shown in red color.

**Table S1**: **Characterization of ORFs in 5553 bp long DNA fragment obtained from cellulase positive clone.**  The table depicts the functional assignment of each ORF along with their e-values and accession number. Cellulase encoding ORF was predicted to belong to GH5 family with high confidence.

| **ORF** | **GC content (%)** | **Length of ORF** | **Number of amino acids** | **Function based on conserved domains** | **e-value** | **Accession number** |
| --- | --- | --- | --- | --- | --- | --- |
| 1 | 53 | 308bp | 101 | Major Facilitator Superfamily (secondary transporters that includes uniporters, symporters, and antiporters) | 5.75e-17 | pfam13347 |
| 2 | 53 | 2436bp | 811 | Glycosyltransferase family 36 (cellobiose phosphorylase (EC:2.4.1.20), cellodextrin phosphorylase (EC:2.4.1.49), chitobiose phosphorylase (EC:2.4.1.-) | 2.48e-43 | pfam06165 |
| 3 | 51 | 903bp | 300 | AGE domain (N-acyl-D-glucosamine 2-epimerase domain) | 3.58e-33 | cd00249 |
| 4 | 51 | 1014bp | 338 | Cellulase (glycosyl hydrolase family 5) | 2.19e-61 | pfam00150 |

**GH5 *Bacteroides* sp. 14(A) WP 025725786.1**

**GH5 *CandidatusBacteroides timonensis* WP 052356300.1**

**GH5 *Bacteroides cellulosilyticus* EEF89984.1**

**Putative cellulase *Bacteroides cellulosilyticus* CDB72026.1**

**GH5 *Bacteroides cellulosilyticus* WP 034753245.1**

**GH5 *Bacteroides stercorirosoris* WP 025834437.1**

**GH5 *Verrucomicrobiae* bacterium DG1235 EDY84973.1**

**GH5 *Flavobacterium beibuense* KGO79203.1**

**GH5 *Flavobacterium beibuense* WP 052123449.1**

**GH5 *Flavobacterium soli* WP 026705711.1**

**GH5 *Flavobacterium* sp*.* AED WP 039109544.1**

**GH5 *Flavobacterium* sp. 83 WP 035673588.1**

**GH5 *Paludibacter propionicigenes* WP 013445319.1**

**Novel GH5 Endoglucanase Cel5R AND74761**

**GH5 *Algoriphagus terrigena* WP 026967447.1**

**GH5 *Bacteroides* sp*.* 2 1 22 EFF55241.1**

**GH5 *Bacteroides ovatus* ATCC 8483 EDO11417.1**

**GH5 *Bacteroides ovatus* WP 004302609.1**

**GH5 *Bacteroides* sp. D2 WP 009001131.1**

**uncharacterized protein *Bacteroides* sp. CAG:20 CCX95073.1**

**GH5 *Barnesiella intestinihominis* WP 008862225.1**

**GH5 *Prevotella* sp. S7 MS 2 WP 036898910.1**

**hypothetical protein *Prevotella paludivivens* WP 018463217.1**

**endoglucanase *Prevotella* sp*.* CAG:732 CDD17780.1**

**GH5 *Prevotella* sp*.* AGR2160 WP 028909656.1**

**GH5 *Prevotella buccae* WP 004345518.1**

**GH5 *Prevotella denticola* WP 048799426.1**

**GH5 *Prevotella* sp*.* MSX73 EJP31037.1**

**0.05**

**Fig S2. Phylogenetic tree construction by Neighbor joining method.** All homologous protein sequences along Cel5Rα sequence were aligned using ClustalW alignment tool. This alignment was used as an input to generate unrooted Neighbor-joining tree [model: Poisson-based; bootstrap: 1000] using MEGA6.06 software. Tree entry information: Pfam hit GH family; organism name followed by accession number.

**Table S2: List of primers used in study**

| **Primer name** | **Sequence (5’-3’)** |
| --- | --- |
| Cel5R F | AATATA**CATATG**AAGAAAAACTCAATCATTCTC |
| cel5R R | AATATA**GGATCC**TCAGATATCCGGGTTTTCATC |
| cel5R Δ27 F | AATATA**CATATG**GAAAACAACAGGAAAACGGACTA |
| C65A F | CGATTGGAAA**GCG**ACGGTCGTCAG |
| C65A R | CTGACGACCGT**CGC**TTTCCAATCG |
| C90A F | GAATTTGCCTTACAG**GCG**ATCACCCCTG |
| C90A R | CAGGGGTGAT**CGC**CTGTAAGGCAAATTC |
| C231A F | CGTATCGGAG**GCG**GGCGGCTC |
| C231A R | GAGCCGCC**CGC**CTCCGATACG |
| C273A F | GAACGAAACC**GCG**TCCATGCTGCTC |
| C273A R | GAGCAGCATGGA**CGC**GGTTTCGTTC |

**1LF1**  ---------- ---------- ---------- ---------- DDYSVVEEHG QLSISNGELV NERGEQVQLK 30

**3PZT**  ---------- -----MGSSH HHHHHSSGLV PRGSHMASAA GTKTPVAKNG QLSIKGTQLV NRDGKAVQLK 55

**1EGZ**  ---------- ---------- ---------- ---------- -SVEPLSVNG NKIYAGEKAK SFAGNSLFWS 29

**1TVN**  ---------- ---------- ---------- ---------- -AVEKLTVSG NQILAGGENT SFAGPSLFWS 29

**2CKS**  ---------- ---------- ---------- ---------- -TGTPVERYG KVQVCGTQLC DEHGNPVQLR 29

**4HTY**  XGSSHHHHHH SSGLVPRGSH XDNAWETTSG WWNASDIPAF DKSKITRQLP LIKVEGNRFV DEQGKTIVFR 70

**1QI2**  ---------- ---------- ---------- ---------- DNDSVVEEHG QLSISNGELV NERGEQVQLK 30

**5I2U**  -------MKK NSIILTLVLF VIVALSCTGS SKKTENNRKT DYRSIVAQNG RLQVIGTQLS NEKGEPVVLR 63

**1LF1**  GM-SSHGLQW YGQFVNYESM KWLRDDWGIT VFRAAM--YT SSGGYI--DD PSVKEKVKET VEAAIDLGIY 95

**3PZT**  GI-SSHGLQW YGEYVNKDSL KWLRDDWGIT VFRAAM--YT ADGGYI--DN PSVKNKVKEA VEAAKELGIY 120

**1EGZ**  NN-GWGGEKF Y----TADTV ASLKKDWKSS IVRAAMGVQE S-GGYL-QDP AGNKAKVERV VDAAIANDMY 92

**1TVN**  NT-GWGAEKF Y----TAETV AKAKTEFNAT LIRAAIGHGT STGGSLNFDW EGNMSRLDTV VNAAIAEDMY 94

**2CKS**  GM-STHGIQW FDHCLTDSSL DALAYDWKAD IIRLSMYIQE DGYETN---P RGFTDRMHQL IDMATARGLY 95

**4HTY**  GVNISDPDKI DKDKRFSKKH FEVIRSWGAN VVRVPVHPRA WKERGV---- KGYLELLDQV VAWNNELGIY 136

**1QI2**  GM-SSHGLQW YGQFVNYESM KWLRDDWGIN VFRAAM--YT SSGGYI--DD PSVKEKVKEA VEAAIDLDIY 95

**5I2U**  GA-SLGWHNL WPRFYNKNAV QWLADDWKCT VVRAAMG-LE IEDNYR-ENP EFALQCITPV IESAIENGIY 130

**1LF1**  VIIDWHILSD NDP------- -NIYKEEAKD FFDEMSELYG DYPN-VIYEI ANEPNGS--- -DVTWDNQIK 152

**3PZT**  VIIDWHILND GNP------- -NQNKEKAKE FFKEMSSLYG NTPN-VIYEI ANEPNG---- -DVNWKRDIK 176

**1EGZ**  AIIGWHSHSA EN-------- ---NRSEAIR FFQEMARKYG NKPN-VIYEI YNEPLQ---- --VSWSNTIK 144

**1TVN**  VIIDFHSHEA HT-------- ---DQATAVR FFEDVATKYG QYDN-VIYEI YNEPLQ---- --ISWVNDIK 146

**2CKS**  VIVDWHILTP GDP------- -HYNLDRAKT FFAEIAQRHA SKTN-VLYEI ANEPNG---- --VSWA-SIK 149

**4HTY**  TILDWHSIGN LKSEXFQNNS YHTTKGETFD FWRRVSERYN GINSVAFYEI FNEPTVFNGR LGIATWAEWK 206

**1QI2**  VIIDWHILSD NDP------- -NIYKEEAKD FFDEMSELYG DYPN-VIYEI ANEPNGS--- -DVTWGNQIK 152

**5I2U**  VIIDFHAHNK YT-------- -----EEAKT FFAGMAEKYG EYPN-VIYEI WNEPDY---- --FEWE-EVK 179

* **

**1LF1**  PYAEEVIPVI RDNDPNNIVI VGTGTWS--- ----QDVHHA ADNQLADP-N VMYAFHFYAG THGQNLRDQV 214

**3PZT**  PYAEEVISVI RKNDPDNIII VGTGTWS--- ----QDVNDA ADDQLKDA-N VMYALHFYAG THGQFLRDKA 238

**1EGZ**  PYAEAVISAI RAIDPDNLII VGTPSWS--- ----QNVDEA SRDPINAK-N IAYTLHFYAG THGESLRNKA 206

**1TVN**  PYAETVIDKI RAIDPDNLIV VGTPTWS--- ----QDVDVA SQNPIDRA-N IAYTLHFYAG THGQSYRNKA 208

**2CKS**  SYAEEVIPVI RQRDPDSVII VGTRGWSSLG VSEGSGPAEI AANPVNAS-N IMYAFHFYAA SHRDNYLNAL 218

**4HTY**  AINEEAITII QAHNPKAIAL VAGFNWA--- ----YDLKEA AANPIDRQ-N IAYVSHPYPQ KVGAPYQANW 268

**1QI2**  PYAEEVIPII RNNDPNNIII VGTGTWS--- ----QDVHHA ADNQLADP-N VMYAFHFYAG THGQNLRDQV 214

**5I2U**  TYSEEVIAVI RAIDPDNIIL VGSPHWD--- ----QDLHLV AEDPIRDVSN IMYTMHFYAA THEAWLRDRT 242

* *

**1LF1**  DYALDQ---G AAIFVSEWGT SA-ATGDGGV FLDEAQVW-- --IDFMDERN LSWANWSLTH KDESSAALMP 276

**3PZT**  NYALSK---G APIFVTEWGT SD-ASGNGGV FLDQSREW-- --LKYLDSKT ISWVNWNLSD KQESSSALKP 300

**1EGZ**  RQALNN---G IALFVTEWGT VN-ADGNGGV NQTETDAW-- --VTFMRDNN ISNANWALND KNEGASTYYP 268

**1TVN**  QTALDN---G IALFATEWGT VN-ADGNGGV NINETDAW-- --MAFFKTNN ISHANWALND KNEGASLFTP 270

**2CKS**  REASEL---- FPVFVTEFGT ET-YTGDGAN DFQMADRY-- --IDLMAERK IGWTKWNYSD DFRSGAVFQP 279

**4HTY**  ERDFGFXADK YPVFATEIGY QRATDKGAHI PVIDDGSYGP RITDYFNSKG ISWVAWVFDP DWSPQLFTDY 338

**1QI2**  DYALDQ---G AAIFVSEWGT SA-ATGDGGV FLDEAQVW-- --IDFMDERN LSWANWSLTH KDESSAALMP 276

**5I2U**  DEAIAK---G IPVFVSECGG SE-ANGDGRL GIEEWKTY-- --VDWMESRK ISWVAWSVSD KNETCSMLLP 304

* *

**1LF1**  GANPTGG-WT EAELSPSGTF VREKIRESAS IPP-- 308

**3PZT**  GASKTGG-WR LSDLSASGTF VRENILGT-- ----- 327

**1EGZ**  DS-------- -KNLTESGKK VKSIIQSWPY KA--- 291

**1TVN**  GGS------- WNSLTSSGSK VKEIIQGWGG ----- 293

**2CKS**  GTCASGGPWS GSSLKASGQW VRSKLQS--- ----- 306

**4HTY**  QTYTPTX--- ------QGEH FRKVXLQDNK ----- 359

**1QI2**  GANPTGG-WT EAELSPSGTF VREKIRESAS ----- 305

**5I2U** RASADGN-WT EDLLKPWGKL TRNSIRNAND ENPDI 338

**Fig S3. Multiple sequence alignment of the GH5 family endoglucanases obtained by Blastp against PDB database showing conserved amino acid residues in Cel5R**. Identical and similar residues are shown by black and grey color respectively. Conserved active site residues (Glu) acting as catalytic nucleophile and general acid/base in GH5 family cellulases are shown in red boxes. Other conserved active site residues are marked with asterisk (*). The position of cysteine residues are marked with arrows. **1LF1 (**Alkalophilic *Bacillus Sp*., identity-44%); **3PZT** (*Bacillus Subtilis* 168, identity-44%); **1EGZ (***Erwinia Chrysanthemi*, identity-41%)**; 1TVN (***Pseudoalteromonas Haloplanktis*, identity-38%**); 2CKS (***Thermobifida Fusca*, identity-36%**); 4HTY (**Metagenome derived cellulase, identity-29%**); 1QI2 (***Bacillus Agaradherans*, identity-44%**); 5I2U (Cel5R).**
